# Design and implementation of a comprehensive management platform for drilling engineering

**DOI:** 10.1371/journal.pone.0343700

**Published:** 2026-02-26

**Authors:** Yaosen Du, Yiyong Yang, Xiaolong Wu, Pengju Gao, Hanchen Ma

**Affiliations:** 1 School of Engineering and Technology, China University of Geosciences, Beijing, China; 2 Institute of Exploration Techniques, Chinese Academy of Geological Sciences（CAGS), Langfang, Hebei, China; 3 Innovation Base for Automatic and Intelligent Drilling Equipment, Geological Society of China, Langfang, Hebei, China; 4 State Key Laboratory of Automotive Safety and Energy, Tsinghua University, Beijing, China; Wadia Institute of Himalayan Geology, INDIA

## Abstract

To enhance the efficiency, safety, and data accuracy of drilling engineering, this study developed an integrated business management platform for drilling engineering grassroots units based on the Business Model Driven (BMD) approach. The platform is built on a “five horizontal, three vertical” cloud computing architecture, establishing a five-layer system from the infrastructure layer to the user layer horizontally, and supported by standard specifications, safety, and maintenance systems vertically, enabling collaboration across multiple business scenarios and data integration. Currently, four major modules with over 20 functionalities have been developed, supporting applications such as task coordination, engineering supervision, data analysis, and accident handling. Operational results demonstrate that the platform effectively promotes integrated management of drilling engineering through real-time data sharing, full-process quality control, and intelligent decision-making, thereby enhancing operational quality and safety, reducing accident risks, and providing critical technological support for the digital transformation and upgrading of the drilling industry.

## Introduction

Drilling operations are not only a key means of obtaining geological information and data support, but also an important way to explore deep-earth resources, disasters, and environmental issues. Although drilling engineering has a wide range of applications, its working environment is complex, placing increasingly stringent demands on drilling construction technology [1]. With the rapid development of social technology, drilling engineering is constantly advancing toward the exploration of deeper and more complex geological conditions. Against this backdrop, it is necessary to leverage emerging technologies to improve drilling efficiency, ensure drilling safety, and achieve green economic production goals.

As China’s mineral resource exploration expands into deeper and more complex geological formations, the demand for the development of unconventional oil and gas resources is gradually increasing. Drilling operations face multiple challenges, including increased operational difficulty, rising costs, and heightened safety and environmental protection requirements. For example, in offshore drilling operations, mitigating operational risks and environmental threats is a critical prerequisite for ensuring the safety of both personnel and the environment. Wu et al. [2] to enhance the safety of offshore oil drilling, this study focuses on the framework of “risk assessment – operational risk management strategies – safety evaluation strategies” to conduct an in-depth analysis and propose risk control measures and optimization recommendations for offshore well operations. This safety management strategy can achieve optimal results for single operational entities; however, drilling projects often involve multiple operations. Therefore, Hu et al. [3] breaking away from the “1-1 joint operation” model, this study summarizes operational experiences from three drilling platforms and two joint production operation platforms at a certain oilfield in the Bohai Sea, China, and proposes an effective multi-facility joint operation management model to keep risks within acceptable limits. As operational conditions change, the operational parameters and processes for drilling projects also vary. The selection of drilling processes and data recording can efficiently allocate resources to enhance drilling efficiency. Based on this, Liu et al. [4] proposed that adequate preparations should be made prior to drilling to address various unexpected situations that may arise during drilling. However, this can only be achieved through process selection and data recording. In similar circumstances in the future, manual decision-making and recording will still be required.

With the development and application of electrical automation and artificial intelligence, automated control of drilling platforms has also emerged [5]. Davoodi et al. [6] was used literature metrology analysis to study the current status and future research directions of artificial intelligence in German drilling engineering. At the same time, some researchers have conducted research on specific topics, such as Hao et al [7] was proposed a data-driven directional drilling simulation environment, which takes into account manual operations, environmental changes, and expert operational experience data. The effectiveness of this strategy was verified through case studies. In the operation of drilling systems, operational safety and data collection are equally important, and together they form the core support for ensuring the efficiency and stability of engineering projects. Neither can be neglected. Xiang et al [8] was proposed a full-scale deepwater drilling system simulation model, which optimizes drilling parameters based on the relationship between drilling environment data changes and drilling platform energy losses. This workflow enables real-time optimization of drilling data and reduction of energy losses during the drilling process. The model not only enables comprehensive simulation analysis of offshore drilling blocks but also addresses the challenges of virtual drilling simulation. Ding et al [9]was proposed a mathematical model for intelligent and comprehensive optimization of drilling operations. This model can automatically optimize key data such as platform deployment, scale, quantity, and type during cluster drilling processes. Additionally, some scholars have conducted research from the perspective of comprehensive management of the entire drilling process, such as Liu et al [10] designed and developed a comprehensive management system (IIMS) that includes well site data management, office planning, and cost control, tailored to the characteristics of drilling operations. Wang et al. [11] developed the Intelligent Planning Integrated Office Management System (IPIOMS) to address a variety of functional requirements, including drilling project operations management, statistical queries, and logistics support.

In summary, the field of drilling engineering informatization has seen the emergence of multiple solutions, which can be categorized into several major paradigms based on their core objectives: operation monitoring platforms centered on real-time perception and control [12], industry cloud portals focused on data aggregation and sharing [13], specialized database systems built around information standardization [14], and intelligent decision support systems driven by algorithmic models [15]. While these paradigms have achieved notable success within their respective scopes, they also reveal common limitations: functional dimensions are often narrow, and management processes remain fragmented. A monitoring platform alone can hardly support strategic resource allocation; a data portal cannot fully address the quality control loop of specific projects; and a decision support system detached from systematic management and a robust data foundation becomes little more than a “castle in the air.” Therefore, drilling engineering management urgently requires a new integrated management model capable of bridging these paradigms. Its core features should be “business-oriented, data-driven, intelligence-empowered, and full-cycle covering.” The present study is an exploration of this new paradigm. The comprehensive management platform developed herein is not intended to replace but to integrate and enhance existing systems. Through a unified business model and architecture, it aims to address the pain points of broken management loops and short data value chains.

To this end, this paper takes the comprehensive management of drilling engineering as the research object, conducts the system design and functional design of the comprehensive management platform for drilling engineering, and develops the system platform for specific engineering cases and applies it in practice. This platform can not only break the serious data silos formed by the scattered storage and difficult acquisition of data, but also avoid the lag and errors caused by manual recording of key parameters such as drilling speed, torque, and mud performance. At the same time, the system can assist the management in rationally allocating resources and making the best decisions, reducing accident risks, and ultimately achieving the goals of improving drilling efficiency, enhancing engineering quality, and ensuring safe production.

## System design

### System architecture design

**Fig 1:**
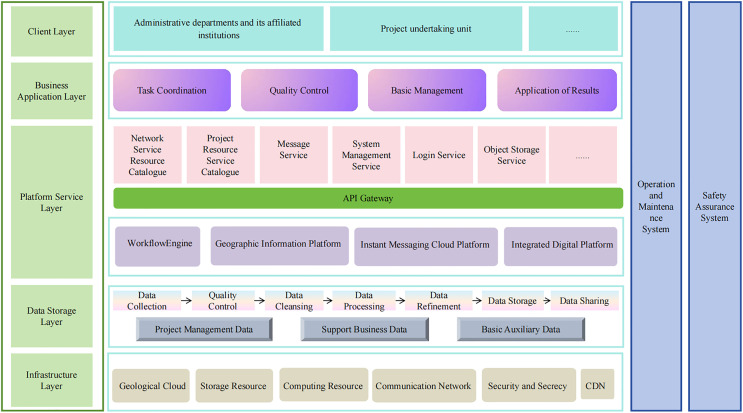
Overall architecture analysis of the comprehensive management platform for drilling engineering.

The construction of the comprehensive management platform for drilling engineering follows a microservice technology architecture and adopts a layered construction approach. Based on user and business requirements and application scenario analysis, the platform adopts a “five horizontal and three vertical” cloud computing structure to build a clear, unified, and efficient system architecture. The “five horizontal” layers include the infrastructure layer, data storage layer, platform service layer, business application layer, and user layer. The “three verticals” include the standards and specifications system, the security assurance system, and the operation and maintenance system. The overall architecture design is shown in Fig 1.

#### Hierarchical structure analysis.

1. Infrastructure layer;

The infrastructure layer provides the basic operating environment for the system platform and is located at the bottom of the entire logical structure. It mainly includes computing resources, storage resources, network resources, etc.

1. Data storage layer;

For drilling engineering data, we’re bringing together, cleaning up, and managing all the existing drilling engineering data across the country. After gathering, checking, cleaning, and processing the data, we've got a basic map data foundation that we’re storing and managing. Then, depending on the data format, we’ll share the data using the right methods.

1. Platform service layer;

Based on the drilling map data foundation, we comprehensively consider national resources and, based on a three-dimensional geographic information platform, provide basic platform capabilities and service capabilities to support the construction of various upper-level business applications.

1. Business application layer;

Based on the capabilities provided by the platform services, we have developed business applications that meet the needs of drilling engineering. The overall project construction mainly includes functions such as task coordination, quality control, basic management, and application of results, enabling interactive display, discussion, prediction, tracking, analysis, and scientific decision-making of drilling engineering data.

1. User layer;

The primary users are professionals and technical personnel involved in geological survey drilling engineering-related businesses in China. The user layer serves as a “bridge” connecting system terminals and system functions, with its interaction interface accessible via various terminal devices such as browsers or mobile devices [16].Chinese geological survey drilling engineering-related business and technical personnel can submit requests to the business application layer through the service window at this layer, or process user requests at other layers.

#### System architecture analysis.

1. Standardization system;

Based on compliance with relevant national, industry, and regional technical standards and specifications in the drilling field, this involves systematic design and development tailored to the practical needs of drilling engineering projects, including system standards and specifications, data standards and specifications, and business process guidelines.

1. Operation and maintenance system;

The operation and maintenance system covers infrastructure monitoring, application monitoring, centralized alarms, and operation and maintenance management. The construction of the operation and maintenance system organizational structure is the foundation of the operation and maintenance system. It comprehensively considers the actual situation and takes into account the establishment of operation and maintenance-related positions and the allocation of responsibilities for each position.

1. Security system;

The security assurance system must strictly implement non-confidential network security level protection requirements, taking a management and technical approach to build an advanced, practical, comprehensive, and reliable multi-layered protection architecture, thereby forming a comprehensive information security assurance system.

### Main functional design

#### Platform functionality implementation.

To meet the flexible and diverse needs of different users, it is necessary to analyze and design the functions of the comprehensive drilling engineering management app based on the technical approach and architecture of this platform, enabling the upload, query, and browsing of drilling engineering data. Different permissions are set according to user levels, and the platform supports viewing and handling simple tasks on mobile devices, such as checking project progress, basic project information, drilling team information, and expert information.

1. Task coordination;

Provides nationwide distribution, key drilling index statistics, drilling project lists, drilling project statistics, expert statistics, and construction team statistics.

1. Engineering supervision;

Provides drilling engineering inquiries, engineering lists, engineering tracking, supervision details, information reporting, and other content displays and functional operations.

1. Data query;

Provides query functions for raw data, document data, and standard specifications.

1. Drilling team;

Provides information query, information editing, information deletion, information listing, drilling team capability assessment, and other content display and functional operations.

1. Expert database;

Provides expert information queries, information editing, information deletion, expert selection, and other content display and functional operations.

1. Technical training;

Provides technical training management functions, including training records, sending and receiving notifications, results management, and other content displays and functional operations.

1. Specialized maps;

Provides a special map module, including map list, query, deletion, and other content display and functional operations.

1. Accident handling analysis;

Provides an accident handling analysis module, including a comprehensive overview of large-scale model resources, resource lists, and interactive dialogue modules, along with content display and functional operations.

1. Drilling process analysis;

Provides a drilling process analysis module, including a large model resource overview, resource list, dialogue interaction module, and other content displays and functional operations.

#### Interoperability with other platforms.

**Fig 2:**
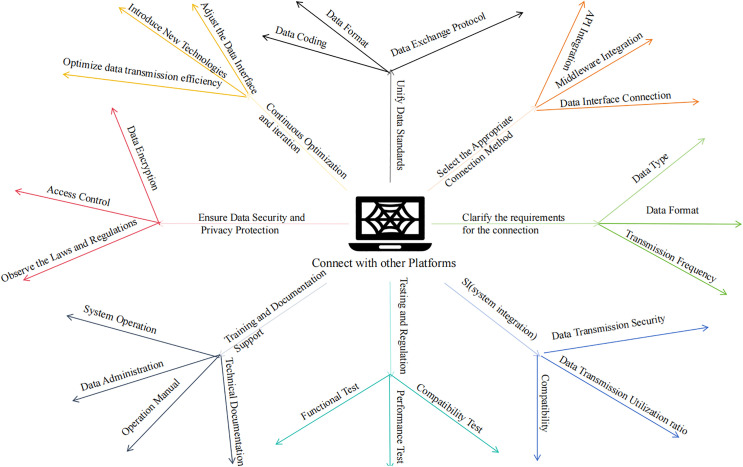
Design of interface requirements between the comprehensive management platform for drilling projects and other platforms.

In practical engineering applications, drilling engineering serves as a technical means of penetrating geological strata through mechanical methods to obtain underground information or facilitate resource development. Its core value lies in “connecting the surface with the subsurface,” thereby forming a closely interdependent collaborative relationship with engineering projects that rely on underground information, underground construction, or resource development. As a result, engineering applications inevitably require the integration of independent platforms with other platforms. Before enabling data sharing and integration between different systems, it is essential to first clarify the specific integration requirements. Secondly, when integrating different systems, it is crucial to select an appropriate data sharing method to ensure rapid data exchange. Additionally, when integrating different platforms, it is necessary to meet the eight key requirements outlined in Fig 2.

## System application effect display

### Project overview

#### Task coordination.

Based on the drilling project information that has been entered and maintained, the system supports statistics on the total number of projects, completed projects, ongoing projects, pending projects, overall progress, and project warnings. It also supports statistics and switching between pie charts, bar charts, and line charts. From the user's perspective of the comprehensive drilling project management platform, it can support the overall work deployment of the new round of mineral exploration breakthrough strategic action through a “single map overview.” This functionality visually presents the distribution of national drilling projects on a map, and information such as the project level, execution year, funding source, completion status, innovative achievements, and evaluation grade for each project can be obtained from the comprehensive management cloud platform, while ensuring data sharing.

#### Drilling project.

After the launch of this platform, the main achievements in drilling engineering can be seen in special maps, accident handling analysis, and drilling process analysis.

**Fig 3:**
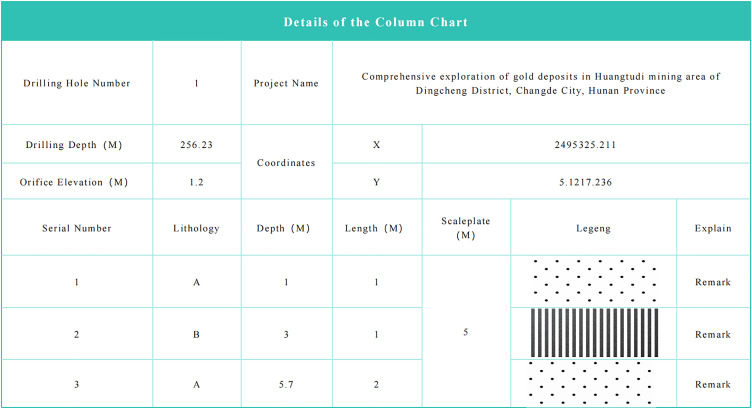
Drill hole column chart.

1. Specialized maps;

Specialized maps are used for drilling engineering borehole maintenance and chart display. Based on the maintenance borehole attributes and borehole mineral type legends, borehole columnar charts are generated, as shown in Fig 3. The system supports the automatic generation of professional borehole columnar charts and integrates them for display in list form.

1. Accident handling analysis;

As a key feature of the platform, this module can quickly analyze historical accident data. By inputting parameters such as accident type and geological conditions, it uses a large model to generate the optimal solution, simultaneously providing comprehensive information on accident causes and response measures. It also supports full lifecycle management of accident-related data, providing data support for subsequent projects and assisting in efficient decision-making to handle accidents such as well collapses and drill bit burials.

1. Drilling process analysis;

Similar to the accident handling and analysis function, this function accompanies the entire drilling process. It can import and maintain process information such as wellbore design and equipment selection, providing a data foundation for analysis. At the same time, it can evaluate parameters and match preliminary design plans based on historical data and input exploration targets and geological conditions through a large model, assisting in the selection of drilling processes.

### Quality control

#### Project supervision and tracking.

**Fig 4:**
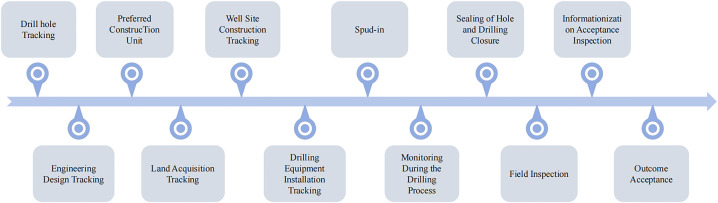
Quality Control module of the comprehensive management platform for drilling engineering.

Engineering supervision work requires users to photograph and upload the on-site conditions of drilling projects during the “pre-drilling, drilling, and post-drilling” phases in accordance with requirements and standards to the engineering supervision function layer to assist the system in identifying data. During this phase, the following conditions require engineering supervision and tracking: borehole location tracking, engineering design tracking, land acquisition tracking, wellsite construction tracking, drilling equipment installation tracking, drilling commencement supervision, drilling process supervision, completion and capping supervision, field acceptance supervision, information technology acceptance supervision, and final acceptance supervision. The simplified process is shown in Fig 4.

#### Acceptance of engineering results.

Project acceptance is a critical stage in the entire lifecycle of drilling projects. It not only serves as a comprehensive inspection of project outcomes but also acts as the core means of ensuring the quality, safety, compliance, and sustainability of drilling projects. In this module, the platform will conduct project acceptance based on photos, standards, opinion letters, and other materials uploaded by system users. The acceptance process will be led by outstanding review experts selected by the system. The process is illustrated in Fig 4.

### Basic management

#### Drilling team management.

Establish a drilling team database to support comprehensive management and optimization of drilling team information, thereby providing support for the selection of drilling project contractors. This primarily includes functions such as drilling team information import, export, listing, addition, editing, deletion, blacklisting, and capability assessment.

#### Expert reviewer management.

**Fig 5:**
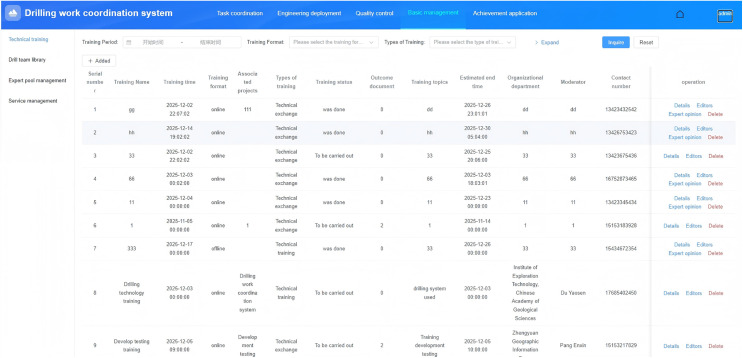
Expert reviewer management.

Establish an expert review database to support comprehensive management and selection of expert information, thereby providing support for review, technical exchange training, and technical consultation at various stages of drilling projects. Its functional design is shown in Fig 5. It mainly includes functions such as importing and exporting expert information, listing, querying, adding, editing, deleting, and blacklisting.

#### Technical guidance and training.

**Fig 6:**
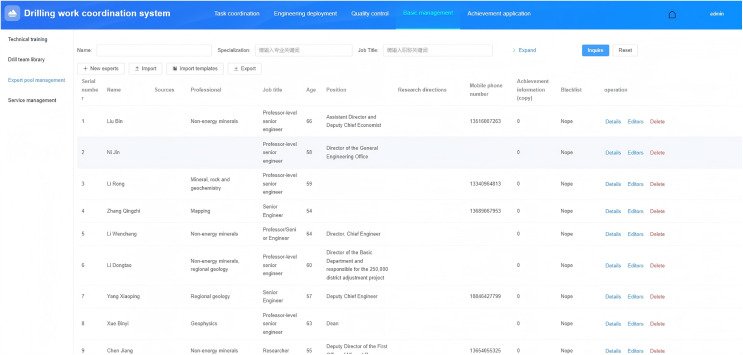
Technical guidance and training.

Technical exchange and training activities focused on drilling technology and challenges are supported by a system that comprehensively manages records of exchanges and training, as well as training outcomes, and issues notifications based on the scope of training participants. The system primarily includes functions such as exchange and training, notification issuance, and training outcome management. The functional design is shown in Fig 6.

### Other management

#### Data management.

**Fig 7:**
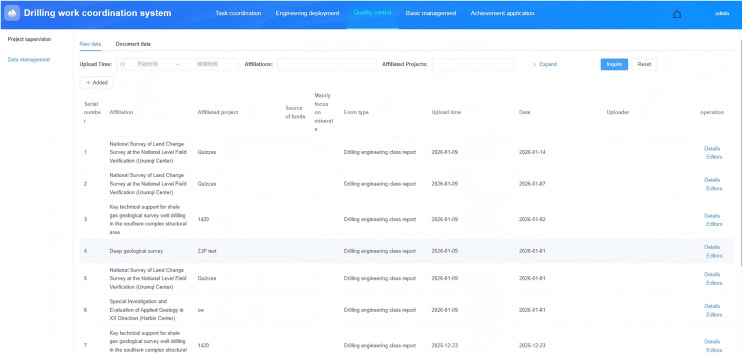
Data Management.

Data management is organized by project to maintain raw data and document data from drilling operations. The overall functional design is shown in Fig 7. Specifically, the management of raw data must support the addition, import, query, export, deletion, and association of raw data such as drilling work shift reports, drilling time logging, core logging, and well logging. Meanwhile, the management of document data must support the addition, import, query, export, deletion, and association of document data such as annual designs, engineering designs, quality inspections, field reports, and results reports.

#### User management.

This feature primarily provides user information management within the platform and is a core module of the system. Administrators can use this feature to create new users, including information such as account availability and expiration dates. At the same time, administrators can create organizational departments and institutions to manage users by organization, including adding, deleting, and modifying organizational information. Organizations are managed in a tree structure and can be divided into multiple levels of parent-child hierarchies.

#### Permission management.

Permission management is a core component of ensuring the security and orderly operation of the platform. Permission management refers to the management of users’ data access permissions. Administrators can grant and revoke users’ access permissions to various types of data, and can also configure data permissions for various maintenance units (e.g., add, delete, or modify data permission information).

#### Log management.

In drilling engineering, the processes involved are complex, the data is sensitive, and collaboration across multiple platforms is required to complete the work. Therefore, in the management platform, log management serves as the core module for recording, storing, and analyzing all operational activities and system states of the platform. Its role spans the entire lifecycle of the system, acting as the “invisible ledger” that ensures the platform’s security, compliance, and traceability. This ledger supports queries for general logs, operation logs, exception logs, and login logs, enabling the comprehensive management platform to achieve its safety and efficiency optimization objectives in terms of risk control and operational value.

#### Comparison of system application effectiveness.

The advanced nature of this platform is manifested in its application scenarios through the capability to address complex, cross-hierarchical management problems. It is designed to tackle the management challenge of quickly responding to local operational anomalies while optimizing global resource allocation in a multi-project concurrent environment:

Under traditional paradigms, relying on systems like [12] can detect anomalies in a single well, but the decision-making and adjustment process requires coordination across different systems, leading to lengthy procedures.

In contrast, this platform enables an endogenous linkage response.When a complex formation warning is triggered via the supervision module for a specific project, this event can be automatically linked to the task coordination module, impacting the global schedule view. Simultaneously, the process analysis module can generate handling suggestions based on historical data, while the team management module can concurrently evaluate and recommend available expert and equipment resources. Managers can access a comprehensive situation overview and issue coordinated commands within a single interface. This compresses the original cross-system, cross-departmental serial workflow into a parallel analysis and decision-making process within the platform, based on a unified data model, significantly enhancing management agility. This demonstrates how the platform, through integrated design, synthesizes capabilities originally dispersed across different paradigm systems into a new, more efficient problem-solving mode.

## Conclusion

The comprehensive drilling engineering management platform developed in this study aims to transcend the single-function paradigm and explore a new pathway toward integrated management. Compared to existing systems that focus solely on operational monitoring, data sharing, information standardization, or intelligent decision-making, the core advancement of this platform lies not in the enhancement of isolated functional points, but in the achievement, for the first time at the system level, of deep vertical integration across strategic planning, operational control, field execution, and intelligent analysis, as well as dynamic horizontal coordination among multiple dimensions such as human resources, equipment, materials, processes, and data. This is realized through the Business Model-Driven (BMD) approach and the five-horizontal, three-vertical architectural framework. The platform provides a systematic digital foundation for addressing the long-standing issues of management fragmentation and data chain disconnection in drilling engineering, thereby promoting a paradigm shift in the industry from developing isolated tools toward building an integrated management operating system.

## Supporting information

S1 File Zttcgl-master.zip. The original code is for APP of the platform.
(ZIP)

S2 File Zttcglweb-master.zip. The original code is for Web of the platform.
(ZIP)
