# Supplementary material for: Design and implementation of a comprehensive management platform for drilling engineering
Source: PLoS One. 2026 Feb 26;21(2):e0343700. doi: 10.1371/journal.pone.0343700 (PMC12944780; doi:10.1371/journal.pone.0343700)
Supplement: S2 File — The original code is for Web of the platform. (ZIP) [file pone.0343700.s002.zip › zttcglweb/public/tables/_井_开固施工记录.htm]

|  |  |  |  |  |  |  |  |  | | 井 |  | | 开固井施工记录 | | | | |  |  |  |  |  |
|  |  | | | | | | | | | | | | | | |  | 日期： | |  | |  |  |
| 井深/m | | | |  | | | | 井径/m | | | |  | | | | 套管下深/m | | |  | | | |
| 水泥返高/m | | | |  | | | | 泥浆密度/g/cm³ | | | |  | | | | 最大井斜/° | | |  | | | |
| 套管串结构 | | | |  | | | | | | | | | | | | | | | | | | |
| 注水泥浆施工计算 | | | | | | | | | | | | | | | | | | | | | | |
| 类别 | | | 环空 | | | | | | 设计水泥浆 | | | | | | 段长/m | | 体积/m³ | | | 附加系数 | | |
|
| 外径/m | | | 内径/m | | | 顶深/m | | | 底深/m | | |
| 套管重合段 | | |  | | |  | | |  | | |  | | |  | |  | | |  | | |
| 裸眼段 | | |  | | |  | | |  | | |  | | |  | |  | | |  | | |
| 套管内 | | |  | | |  | | |  | | |  | | |  | |  | | |  | | |
| 水泥浆总体积/m³ | | | |  | | | | 水泥灰重/t | | | |  | | | | 水灰比 | | |  | | | |
| 水泥浆配方 | | | |  | | | | | | | | | | | | | | | | | | |
| 替浆量计算 | | | |  | | | | | | | | | | | | | | | | | | |
| 替浆量/m³ | | | |  | | | | 后置液/m³ | | | |  | | | | 替泥浆/m³ | | |  | | | |
| 实际固井施工数据 | | | | | | | | | | | | | | | | | | | | | | |
| 注水泥、顶替泵 | | | |  | | | | | | | | 注水泥浆压力/MPa | | | |  | | | | | | |
| 注前置液量/m3 | | | |  | | | | | | | | 注水泥浆量/m3 | | | |  | | | | | | |
| 水泥浆密度/g/cm3 | | | | 最大 | | | |  | | | | 最小 | |  | | | 平均 | | |  | | |
| 注后置液量/m3 | | | |  | | | | | | | | 替钻井液量/m3 | | | |  | | | | | | |
| 替钻井液时间/min | | | |  | | | | | | | | 替浆压力/Mpa | | | |  | | | | | | |
| 使用材料 | | | |  | | | | | | | | | | | | | | | | | | |
| 备注 | | | |  | | | | | | | | | | | | | | | | | | |
| 施工时间 | | | | | | | | | | | | | | | | | | | | | | |
| 下套管时间 | | |  | | | | 至 |  | | | | 下钻杆时间 | | |  | | | 至 |  | | | |
| 辅助时间 | | |  | | | | 至 |  | | | | 循环时间 | | |  | | | 至 |  | | | |
| 注前置液时间 | | |  | | | | 至 |  | | | | 注水泥浆时间 | | |  | | | 至 |  | | | |
| 注后置液时间 | | |  | | | | 至 |  | | | | 替钻井液时间 | | |  | | | 至 |  | | | |
| 填表人： | | |  | | | | 井队负责： | |  | | |  | | | 固井负责： | |  | | |  | | |
|  |  |  |  |  |
