# Supplementary material for: Design and implementation of a comprehensive management platform for drilling engineering
Source: PLoS One. 2026 Feb 26;21(2):e0343700. doi: 10.1371/journal.pone.0343700 (PMC12944780; doi:10.1371/journal.pone.0343700)
Supplement: S2 File — The original code is for Web of the platform. (ZIP) [file pone.0343700.s002.zip › zttcglweb/public/tables/交接班.htm]

|  | | | | | | | | 接班井深（m） | | |  | | 交班井深（m） | |  | | |  |
| 钻井工程班报表 | | | | | | | | 交班机余（m） | | |  | | 本班进尺（m） | |  | | |  |
|  | 钻井队： |  | | 井号： |  | | |  |  |  |  | 班自 |  | 时至 |  | 时 |  |  |
| 时间 | | 工作内容           起下钻、钻进、取心、循环停待、事故处理、 下套管、 设备保养等 | | 机上余尺 | 进尺及岩矿心长度(m) | | | | | 岩心采取率% | 钻进参数 | | | | 泥浆性能 | | |  |
| h:m | |  |
| 自 | 至 | m | 自 | 至 | 进尺 | 岩心长度 | 块数/回次 | 钻压 | 转速 | 排量 | 立压 | 密度 | 滤矢量 | 粘度 |  |
|  |
|  |
| kN | rpm | L/min | MPa | g/cm3 | ml | s |  |
|  |  |  | |  |  |  |  |  |  |  |  |  |  |  |  |  |  |  |
| 钻具组合 | |  | | | | | | | | | | 钻头名称 | 规格（mm） | 长度（m） | 编号 | | 钻具总长（m） |  |
|  |  |  |  | |  |  |
|  |
| 情况说明 | |  | | | | | | | | | | 扩孔器 | 规格（mm） | 长度（m） | 编号 | |  |  |
|  |  |  |  | |  |  |
|  | 记录员： |  |  |  | | 交班班长： |  | |  | | 接班班长： |  | |  | 井队长： |  | |  |
|  |  |  |  |  |
