# Supplementary material for: Design and implementation of a comprehensive management platform for drilling engineering
Source: PLoS One. 2026 Feb 26;21(2):e0343700. doi: 10.1371/journal.pone.0343700 (PMC12944780; doi:10.1371/journal.pone.0343700)
Supplement: S2 File — The original code is for Web of the platform. (ZIP) [file pone.0343700.s002.zip › zttcglweb/public/tables/取芯统计表.htm]

| 取心统计表 | | | | | | | | | |  |  |
| 井号： |  | | |  | | | | | | |  |
| 取心回次 | 取心日期 | 取心井段（m） | 取心长度（m） | 岩心长度（m） | 岩心采取率(%) | 岩心名称 | 岩性描述 | | | 备注 |  |
|  |  |  |  |  |  |  |  | | |  |  |
|  |  |  |  |  |  |  |  | | |  |  |
|  |  |  |  |  |  |  |  | | |  |  |
|  |  |  |  |  |  |  |  | | |  |  |
|  |  |  |  |  |  |  |  | | |  |  |
|  |  |  |  |  |  |  |  | | |  |  |
|  |  |  |  |  |  |  |  | | |  |  |
|  | | | | 记录人： |  | |  | 审核人： |  | |  |
|  |  |  |  |  |  |  |  |  |
