# Supplementary material for: Design and implementation of a comprehensive management platform for drilling engineering
Source: PLoS One. 2026 Feb 26;21(2):e0343700. doi: 10.1371/journal.pone.0343700 (PMC12944780; doi:10.1371/journal.pone.0343700)
Supplement: S2 File — The original code is for Web of the platform. (ZIP) [file pone.0343700.s002.zip › zttcglweb/public/tables/套管柱试压记录表.htm]

| 套管柱试压记录表 | | | | | | | | |
| 井号： |  | | 井型： |  | | 日期： |  |  |
| 固井 | | | | | | | | |
| 固井日期 | |  | 注水泥起止时间 | |  |  | 备注 |  |
| 固井日期 | |  | 注水泥起止时间 | |  |  | 备注 |  |
| 固井日期 | |  | 注水泥起止时间 | |  |  | 备注 |  |
| 试压 | | | | | | | | |
| 试压设备 | |  | | | 试压液 |  | 试压压力MPa |  |
| 试压起止时间 | |  |  | | 合计min |  | 压降Mpa |  |
| 其他 | |  | | | | | | |
| 套管柱 | | | | | | | | |
| 套管类别 | | 外径mm | 钢级 | | 壁厚mm | 螺纹类型 | 长度m | 下深m |
|  | |  |  | |  |  |  |  |
|  | |  |  | |  |  |  |  |
|  | |  |  | |  |  |  |  |
|  | |  |  | |  |  |  |  |
|  |  | 记录： |  | |  | 审核： |  | |
|  |  |  |  |  |  |  |  |  |
