# Supplementary material for: Design and implementation of a comprehensive management platform for drilling engineering
Source: PLoS One. 2026 Feb 26;21(2):e0343700. doi: 10.1371/journal.pone.0343700 (PMC12944780; doi:10.1371/journal.pone.0343700)
Supplement: S2 File — The original code is for Web of the platform. (ZIP) [file pone.0343700.s002.zip › zttcglweb/public/tables/孔深校正记录.htm]

| 孔深校正记录 | | | | | | | | | | | |
| 井号： |  | | | 施工井队： | |  | | | 日期： |  | |
| 校正孔深次数： | | |  | | | 校正孔深原因： | | |  | | |
| 报表记录孔深（m）： | | |  | | | 实际丈量孔深（m）： | | |  | | |
| 孔深误差： | | |  | | | 误差率（‰）： | | |  | | |
| 误差原因： | | |  | | | 误差处理方法： | | |  | | |
|  |  |  | | | | | | | | |  |
| 立根序号 | 根数 | 长度/m | 立根序号 | 根数 | 长度/m | 立根序号 | 根数 | 长度/m | 立根序号 | 根数 | 长度/m |
|  |  |  |  |  |  |  |  |  |  |  |  |
|  |  |  |  |  |  |  |  |  |  |  |  |
|  |  |  |  |  |  |  |  |  |  |  |  |
|  |  |  |  |  |  |  |  |  |  |  |  |
|  |  |  |  |  |  |  |  |  |  |  |  |
|  |  |  |  |  |  |  |  |  |  |  |  |
|  |  |  |  |  |  |  |  |  |  |  |  |
|  |  |  |  |  |  |  |  |  |  |  |  |
|  |  |  |  |  |  |  |  |  |  |  |  |
|  |  |  |  |  |  |  |  |  |  |  |  |
|  |  |  |  |  |  |  |  |  |  |  |  |
|  |  |  |  |  |  |  |  |  |  |  |  |
|  |  |  |  |  |  |  |  |  |  |  |  |
|  |  |  |  |  |  |  |  |  |  |  |  |
|  |  |  |  |  |  |  |  |  | 合计 | |  |
|  |  |  |  |  |  |  |  |  | 方余 | |  |
|  |  |  |  |  |  |  |  |  | 机高 | |  |
|  |  |  |  |  |  |  |  |  | 孔深 | |  |
|  | 记录人： | |  | |  | | | 审核人： | |  | |
|
