# Supplementary material for: Design and implementation of a comprehensive management platform for drilling engineering
Source: PLoS One. 2026 Feb 26;21(2):e0343700. doi: 10.1371/journal.pone.0343700 (PMC12944780; doi:10.1371/journal.pone.0343700)
Supplement: S2 File — The original code is for Web of the platform. (ZIP) [file pone.0343700.s002.zip › zttcglweb/public/tables/封孔登记表.htm]

| 封孔登记表 | | | | | | | |
| 矿区： |  | | 孔号： |  | | 机台： |  |
| 封孔设计 | | | 封孔结果 | | | | |
| 孔身结构 | 封闭位置 | 地质简述及封孔要求 | 封闭位置 | 木塞位置直径及长度 | 封孔材料用量及配方 | 封孔方法 | 备 注 |
|  |  |  |  |  |  |  |  |
| 记录： |  | |  | 审核： |  | |  |
|  |  |  |  |  |  |  |  |
