# Supplementary material for: Design and implementation of a comprehensive management platform for drilling engineering
Source: PLoS One. 2026 Feb 26;21(2):e0343700. doi: 10.1371/journal.pone.0343700 (PMC12944780; doi:10.1371/journal.pone.0343700)
Supplement: S2 File — The original code is for Web of the platform. (ZIP) [file pone.0343700.s002.zip › zttcglweb/public/tables/岩心取样登记表.htm]

| 岩心取样登记表 | | | | | |
| 井号： |  | | | 日期： |  |
| 序号 | 岩心盒号 | 取样位置（m） | 取样层位 | 岩心长（cm） | 备注 |
|  |  |  |  |  |  |
|  |  |  |  |  |  |
|  |  |  |  |  |  |
|  |  |  |  |  |  |
|  |  |  |  |  |  |
|  |  |  |  |  |  |
|  |  |  |  |  |  |
| 取样单位： |  | | | 取样人： |  |
|  |  |  |
