# Supplementary material for: Design and implementation of a comprehensive management platform for drilling engineering
Source: PLoS One. 2026 Feb 26;21(2):e0343700. doi: 10.1371/journal.pone.0343700 (PMC12944780; doi:10.1371/journal.pone.0343700)
Supplement: S2 File — The original code is for Web of the platform. (ZIP) [file pone.0343700.s002.zip › zttcglweb/public/tables/录井日报表.htm]

| 录井日报表 | | | | | | | | | | |
| 井号 | |  | | 层位 |  | 日期 | | |  | |
| 井深(m) | |  | | | 岩性 |  | | | | |
| 回次 | | 进尺(m) | 心长(m) | | 收获率(%) | | 取心井段(m) | | | |
|  | |  |  | |  | |  | | | |
| 钻时(min/m) | | 最大 | 最小 | | 一般 | | 荧光显示长度(m) | | | |
|  |  | |  | |  | | | |
|  | | | | | | | | | | |
| 气测 异常 | 井段(m) | | 全烃(%) | | C1(%) | C2(%) | | C3(%) | | IC4(%) |
|  | |  | |  |  | |  | |  |
| NC4(%) | | IC5(%) | | NC5(%) | 后效(%) | | 持续时间(S) | | |
|  | |  | |  |  | |  | | |
| 备注 | |  | | | | | | | | |
|
|
|
| 填表人： | |  | |  | | | 审核人： | |  | |
| 日  期： | |  | |  | | | 日  期： | |  | |
|  |  | | | | | | | | | |
|  |  |  |  |  |  |  |  |  |  |
