# Supplementary material for: Design and implementation of a comprehensive management platform for drilling engineering
Source: PLoS One. 2026 Feb 26;21(2):e0343700. doi: 10.1371/journal.pone.0343700 (PMC12944780; doi:10.1371/journal.pone.0343700)
Supplement: S2 File — The original code is for Web of the platform. (ZIP) [file pone.0343700.s002.zip › zttcglweb/public/tables/简易水文观测记录表.htm]

| 简易水文观测记录表 | | | | | | | | | | | | | | | | |
| 井号： |  | | 水箱面积（m2）： | |  | | 井口套管尺寸（mm）： | | |  | | 井口套管下入深度（m）： | | |  | |
| 日期 | 班次 | 钻井液消耗量观测 | | | | | | | | | 孔内水位观测（m） | | | | | 孔内情况（溶洞、破碎带、漏水、涌水、掉块、坍塌等深度） |
| 孔深（m） | | 时间（h） | | | 水箱内水位（m） | | | 消耗量（m3/h） | 观测时孔深（m） | 提钻后 | | 下钻前 | |
| 自 | 至 | 自 | 至 | 计 | 原有 | 剩余 | 差值 | 时间 | 水位 | 时间 | 水位 |
|  |  |  |  |  |  |  |  |  |  |  |  |  |  |  |  |  |
|  |  |  |  |  |  |  |  |  |  |  |  |  |  |  |  |  |
|  |  |  |  |  |  |  |  |  |  |  |  |  |  |  |  |  |
|  |  |  |  |  |  |  |  |  |  |  |  |  |  |  |  |  |
|  |  |  |  |  |  |  |  |  |  |  |  |  |  |  |  |  |
|  |  |  |  |  |  |  |  |  |  |  |  |  |  |  |  |  |
|  |  |  |  |  |  |  |  |  |  |  |  |  |  |  |  |  |
|  |  |  |  |
