# Supplementary material for: Design and implementation of a comprehensive management platform for drilling engineering
Source: PLoS One. 2026 Feb 26;21(2):e0343700. doi: 10.1371/journal.pone.0343700 (PMC12944780; doi:10.1371/journal.pone.0343700)
Supplement: S2 File — The original code is for Web of the platform. (ZIP) [file pone.0343700.s002.zip › zttcglweb/public/tables/钻时记录表.htm]

| 钻时记录表 | | | | | | | |
| 井号： |  | | 录井队： |  | | 日期： |  |
| 井深 | 钻达时间 | 停钻时间 | 钻时 | 开泵时间 | 停泵时间 | 迟到时间 | 捞砂时间 |
| （m） | （时：分） | （时：分） | （分） | （时：分） | （时：分） | （时：分） | （时：分） |
|  |  |  |  |  |  |  |  |
|  |  |  |  |  |  |  |  |
|  |  |  |  |  |  |  |  |
|  |  |  |  |  |  |  |  |
|  |  |  |  |  |  |  |  |
|  |  |  |  |  |  |  |  |
|  |  |  |  |  |  |  |  |
|  |  |  |  |  |  |  |  |
|  |  |  |  |  |  |  |  |
|  |  |  |  |  |  |  |  |
|  |  |  |  |  |  |  |  |
|  |  |  |  |  |  |  |  |
|  | | | 记录人： |  |  | 审核人： |  |
|  |  |
