# Supplementary figures and images for: Design and implementation of a comprehensive management platform for drilling engineering
Source: PLoS One. 2026 Feb 26;21(2):e0343700. doi: 10.1371/journal.pone.0343700 (PMC12944780; doi:10.1371/journal.pone.0343700)

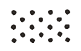

Supplement: S2 File — The original code is for Web of the platform. (ZIP) [file pone.0343700.s002.zip › zttcglweb/public/dizhi/A.png]

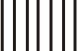

Supplement: S2 File — The original code is for Web of the platform. (ZIP) [file pone.0343700.s002.zip › zttcglweb/public/dizhi/B.png]

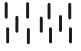

Supplement: S2 File — The original code is for Web of the platform. (ZIP) [file pone.0343700.s002.zip › zttcglweb/public/dizhi/C.png]

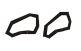

Supplement: S2 File — The original code is for Web of the platform. (ZIP) [file pone.0343700.s002.zip › zttcglweb/public/dizhi/D.png]

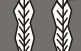

Supplement: S2 File — The original code is for Web of the platform. (ZIP) [file pone.0343700.s002.zip › zttcglweb/public/dizhi/E.png]

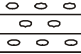

Supplement: S2 File — The original code is for Web of the platform. (ZIP) [file pone.0343700.s002.zip › zttcglweb/public/dizhi/F.png]

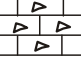

Supplement: S2 File — The original code is for Web of the platform. (ZIP) [file pone.0343700.s002.zip › zttcglweb/public/dizhi/G.png]

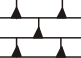

Supplement: S2 File — The original code is for Web of the platform. (ZIP) [file pone.0343700.s002.zip › zttcglweb/public/dizhi/R.png]

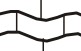

Supplement: S2 File — The original code is for Web of the platform. (ZIP) [file pone.0343700.s002.zip › zttcglweb/public/dizhi/W.png]

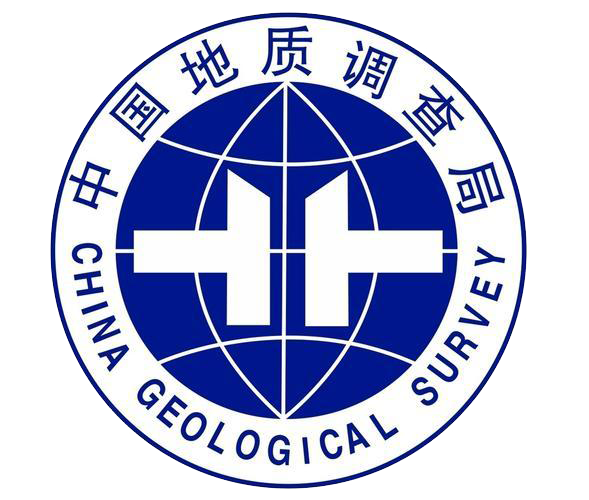

Supplement: S2 File — The original code is for Web of the platform. (ZIP) [file pone.0343700.s002.zip › zttcglweb/public/favicon.png]

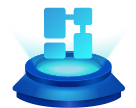

Supplement: S2 File — The original code is for Web of the platform. (ZIP) [file pone.0343700.s002.zip › zttcglweb/src/assets/area.png]

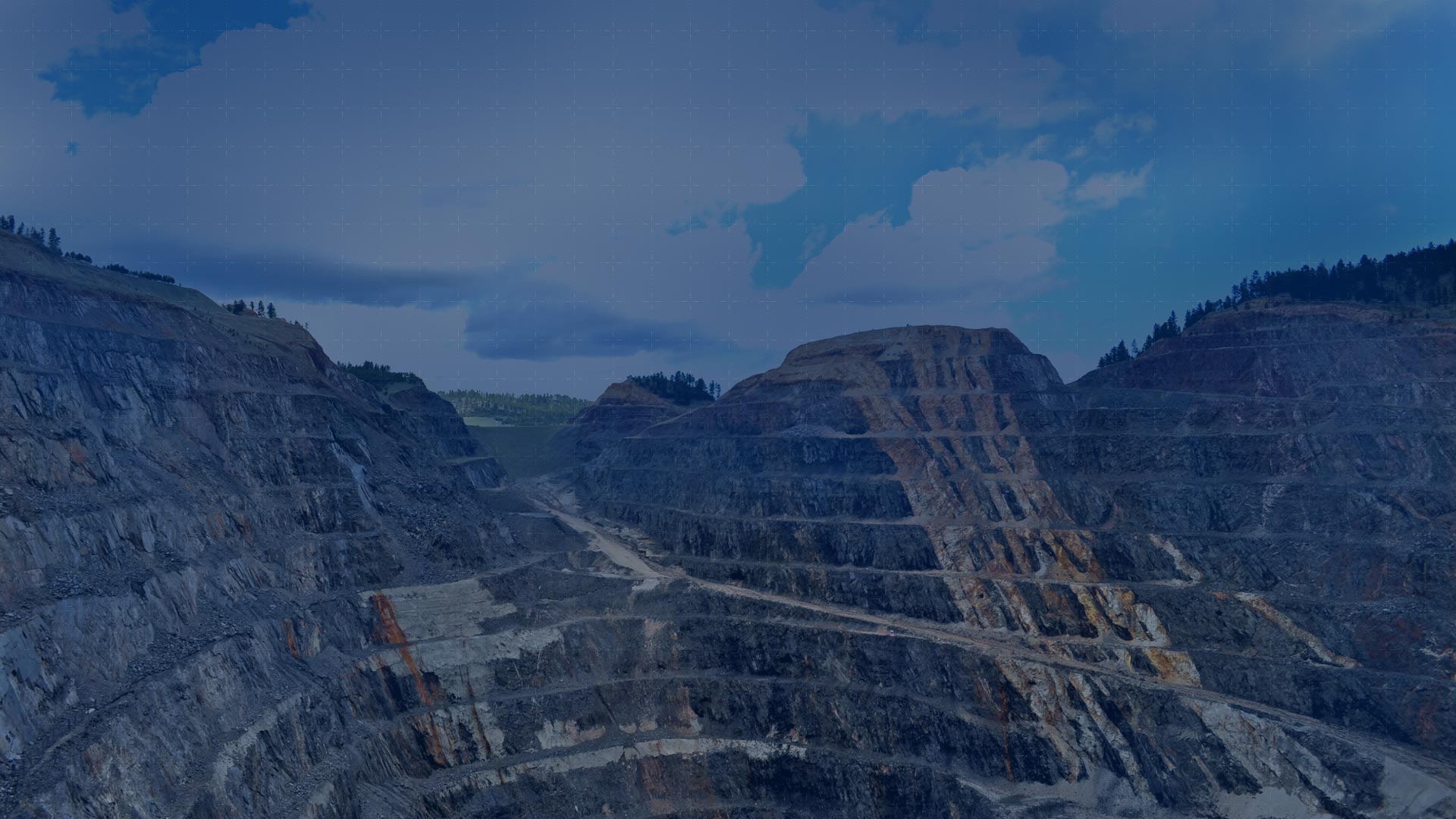

Supplement: S2 File — The original code is for Web of the platform. (ZIP) [file pone.0343700.s002.zip › zttcglweb/src/assets/bg.jpg]

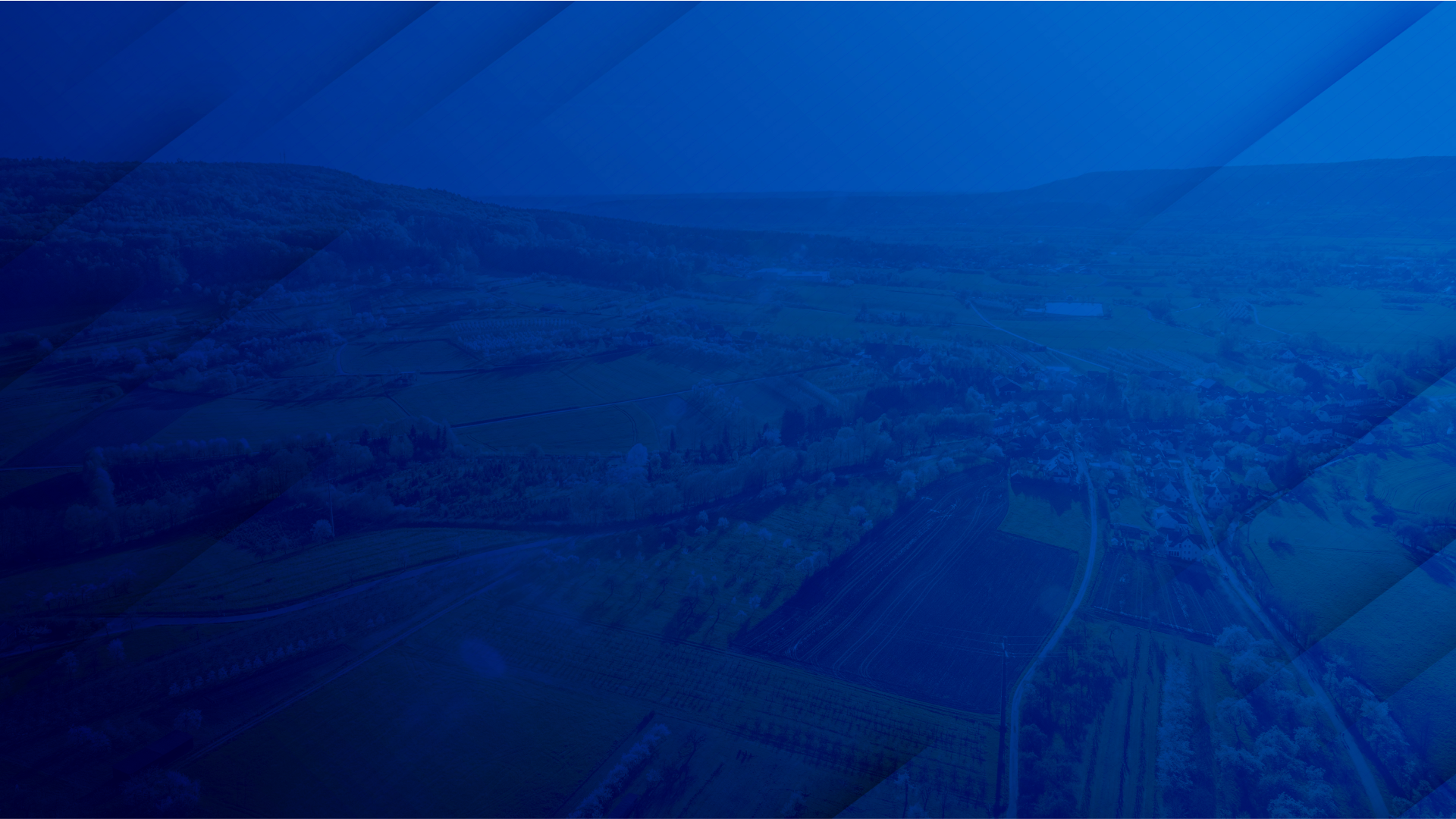

Supplement: S2 File — The original code is for Web of the platform. (ZIP) [file pone.0343700.s002.zip › zttcglweb/src/assets/bg.png]

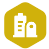

Supplement: S2 File — The original code is for Web of the platform. (ZIP) [file pone.0343700.s002.zip › zttcglweb/src/assets/build.png]

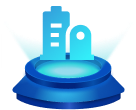

Supplement: S2 File — The original code is for Web of the platform. (ZIP) [file pone.0343700.s002.zip › zttcglweb/src/assets/building.png]

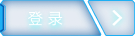

Supplement: S2 File — The original code is for Web of the platform. (ZIP) [file pone.0343700.s002.zip › zttcglweb/src/assets/button.png]

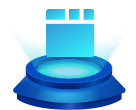

Supplement: S2 File — The original code is for Web of the platform. (ZIP) [file pone.0343700.s002.zip › zttcglweb/src/assets/calendar.png]

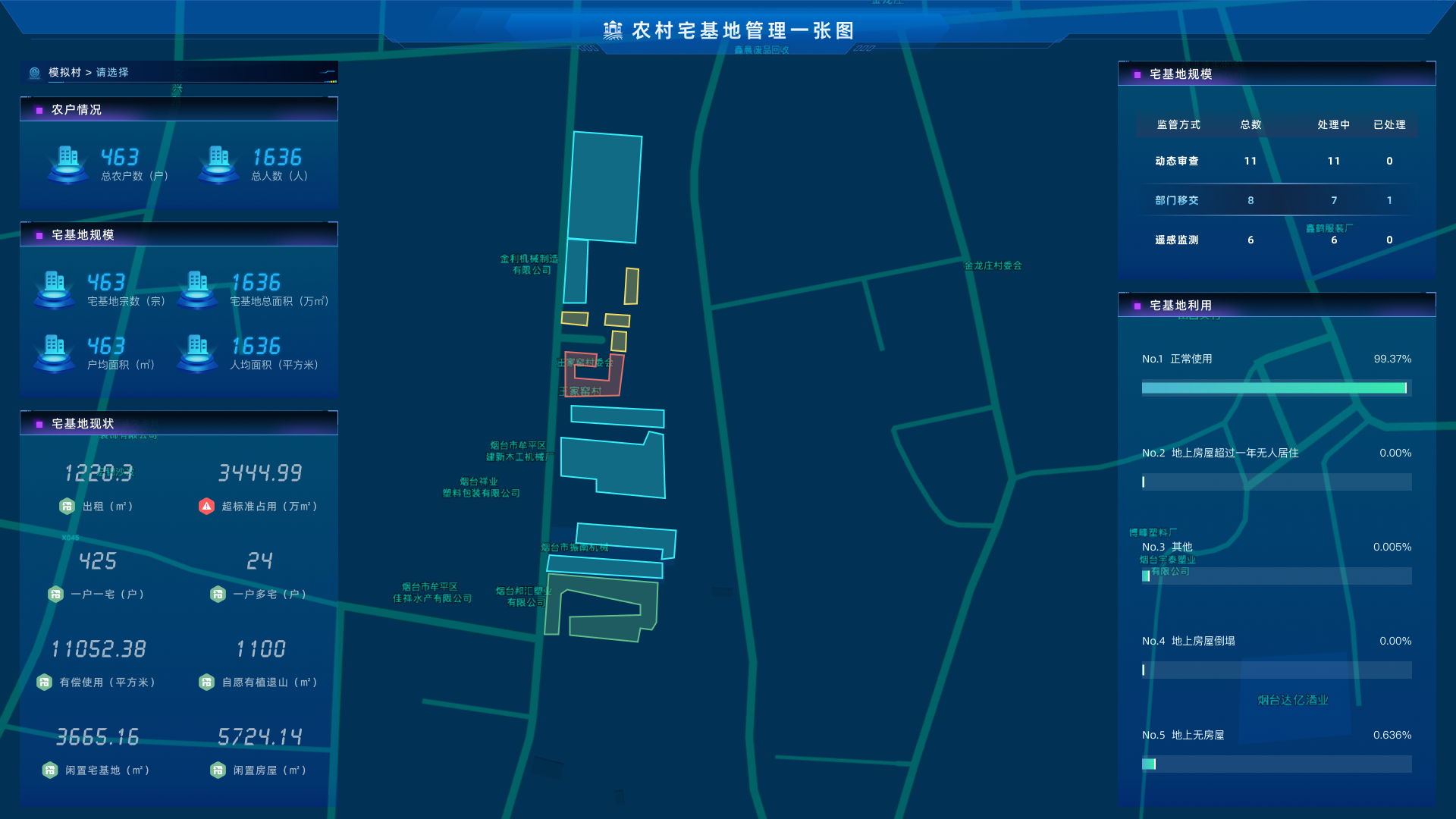

Supplement: S2 File — The original code is for Web of the platform. (ZIP) [file pone.0343700.s002.zip › zttcglweb/src/assets/demo.png]

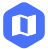

Supplement: S2 File — The original code is for Web of the platform. (ZIP) [file pone.0343700.s002.zip › zttcglweb/src/assets/door-open.png]

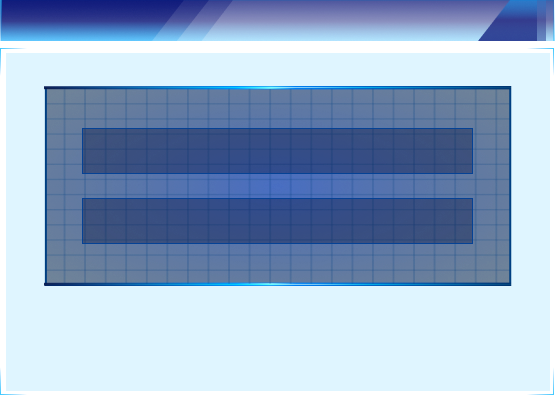

Supplement: S2 File — The original code is for Web of the platform. (ZIP) [file pone.0343700.s002.zip › zttcglweb/src/assets/frame.png]

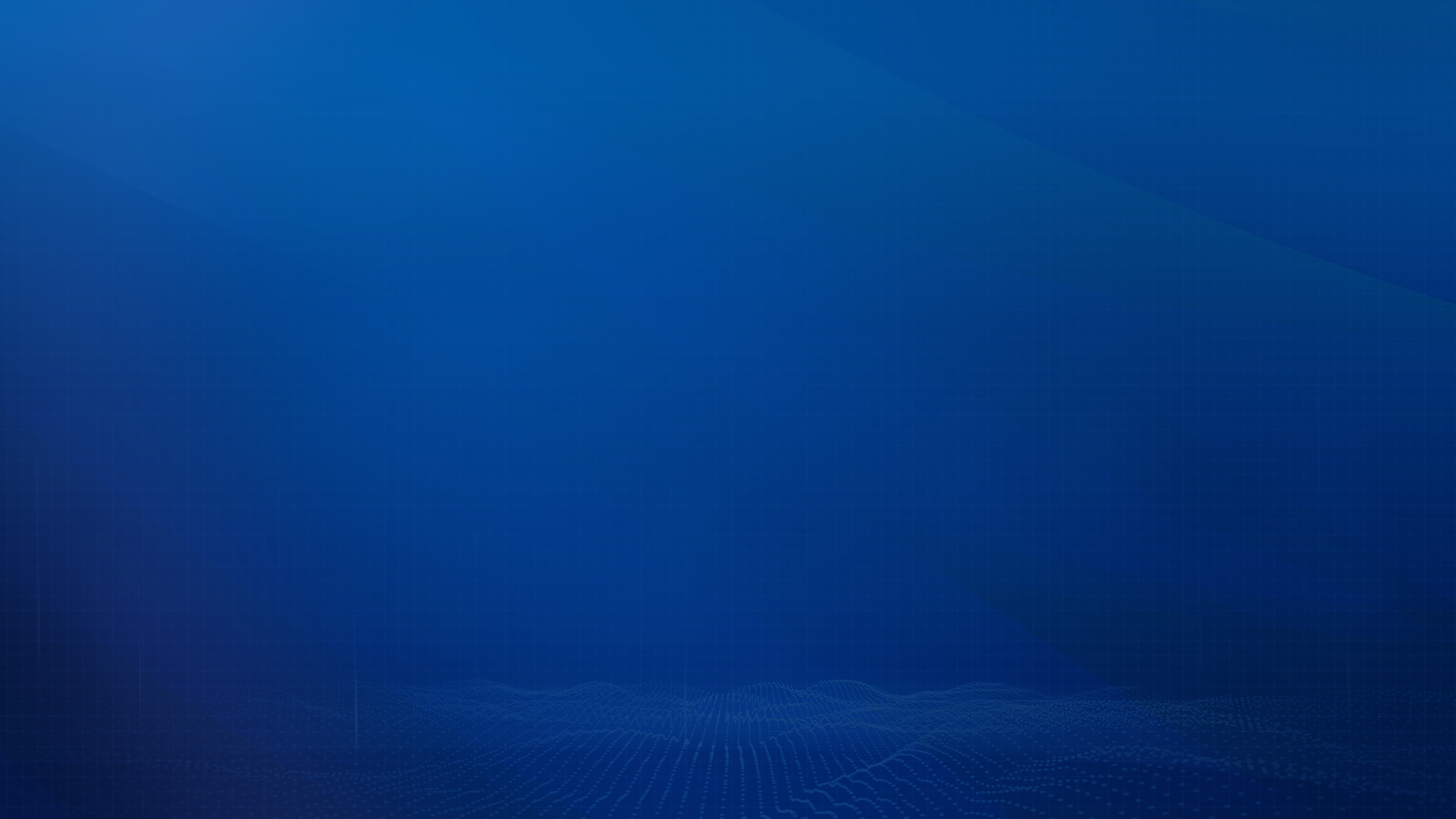

Supplement: S2 File — The original code is for Web of the platform. (ZIP) [file pone.0343700.s002.zip › zttcglweb/src/assets/guide/bg2.png]

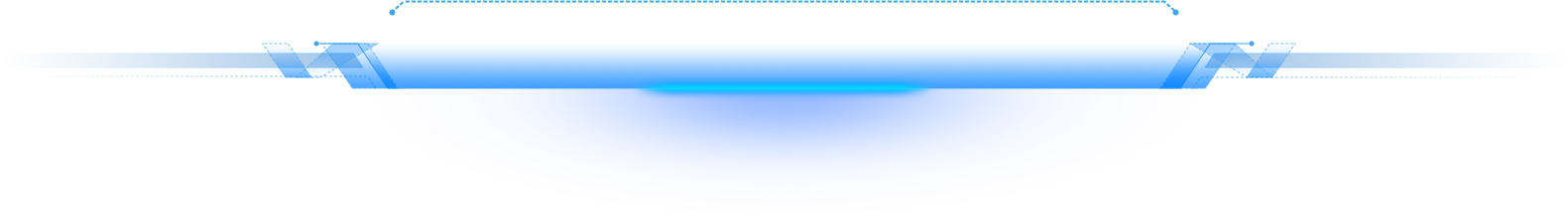

Supplement: S2 File — The original code is for Web of the platform. (ZIP) [file pone.0343700.s002.zip › zttcglweb/src/assets/guide/bg_top.png]

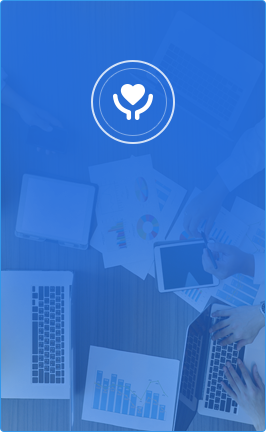

Supplement: S2 File — The original code is for Web of the platform. (ZIP) [file pone.0343700.s002.zip › zttcglweb/src/assets/guide/icon_fuwu.png]

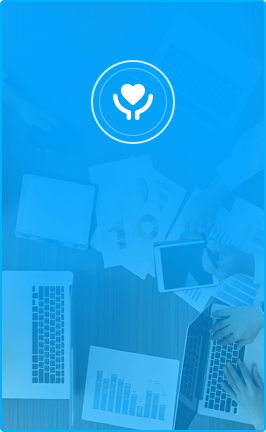

Supplement: S2 File — The original code is for Web of the platform. (ZIP) [file pone.0343700.s002.zip › zttcglweb/src/assets/guide/icon_fuwu_click.png]

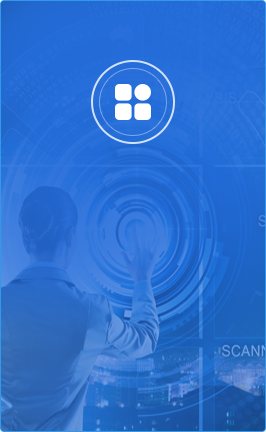

Supplement: S2 File — The original code is for Web of the platform. (ZIP) [file pone.0343700.s002.zip › zttcglweb/src/assets/guide/icon_guanli.png]

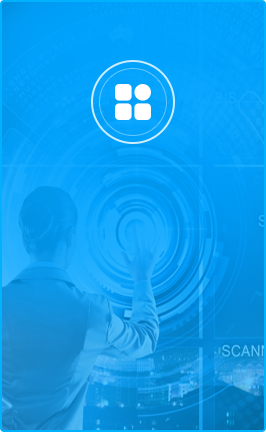

Supplement: S2 File — The original code is for Web of the platform. (ZIP) [file pone.0343700.s002.zip › zttcglweb/src/assets/guide/icon_guanli_click.png]

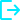

Supplement: S2 File — The original code is for Web of the platform. (ZIP) [file pone.0343700.s002.zip › zttcglweb/src/assets/guide/icon_tuichu.png]

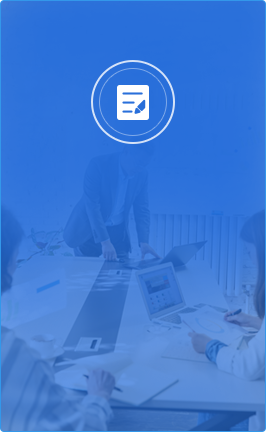

Supplement: S2 File — The original code is for Web of the platform. (ZIP) [file pone.0343700.s002.zip › zttcglweb/src/assets/guide/icon_xuqiu.png]

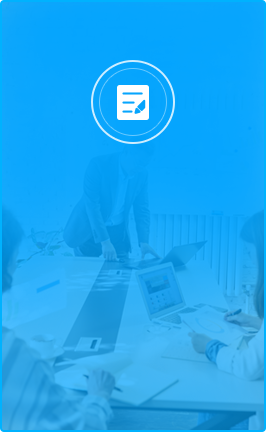

Supplement: S2 File — The original code is for Web of the platform. (ZIP) [file pone.0343700.s002.zip › zttcglweb/src/assets/guide/icon_xuqiu_click.png]

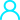

Supplement: S2 File — The original code is for Web of the platform. (ZIP) [file pone.0343700.s002.zip › zttcglweb/src/assets/guide/icon_yonghu.png]

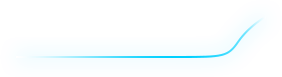

Supplement: S2 File — The original code is for Web of the platform. (ZIP) [file pone.0343700.s002.zip › zttcglweb/src/assets/guide/line_menu.png]

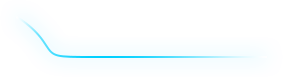

Supplement: S2 File — The original code is for Web of the platform. (ZIP) [file pone.0343700.s002.zip › zttcglweb/src/assets/guide/line_menu2.png]

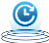

Supplement: S2 File — The original code is for Web of the platform. (ZIP) [file pone.0343700.s002.zip › zttcglweb/src/assets/history.png]

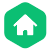

Supplement: S2 File — The original code is for Web of the platform. (ZIP) [file pone.0343700.s002.zip › zttcglweb/src/assets/home-s.png]

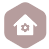

Supplement: S2 File — The original code is for Web of the platform. (ZIP) [file pone.0343700.s002.zip › zttcglweb/src/assets/home-set.png]

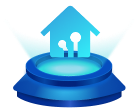

Supplement: S2 File — The original code is for Web of the platform. (ZIP) [file pone.0343700.s002.zip › zttcglweb/src/assets/home.png]

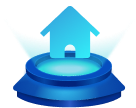

Supplement: S2 File — The original code is for Web of the platform. (ZIP) [file pone.0343700.s002.zip › zttcglweb/src/assets/house.png]

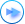

Supplement: S2 File — The original code is for Web of the platform. (ZIP) [file pone.0343700.s002.zip › zttcglweb/src/assets/icon.png]

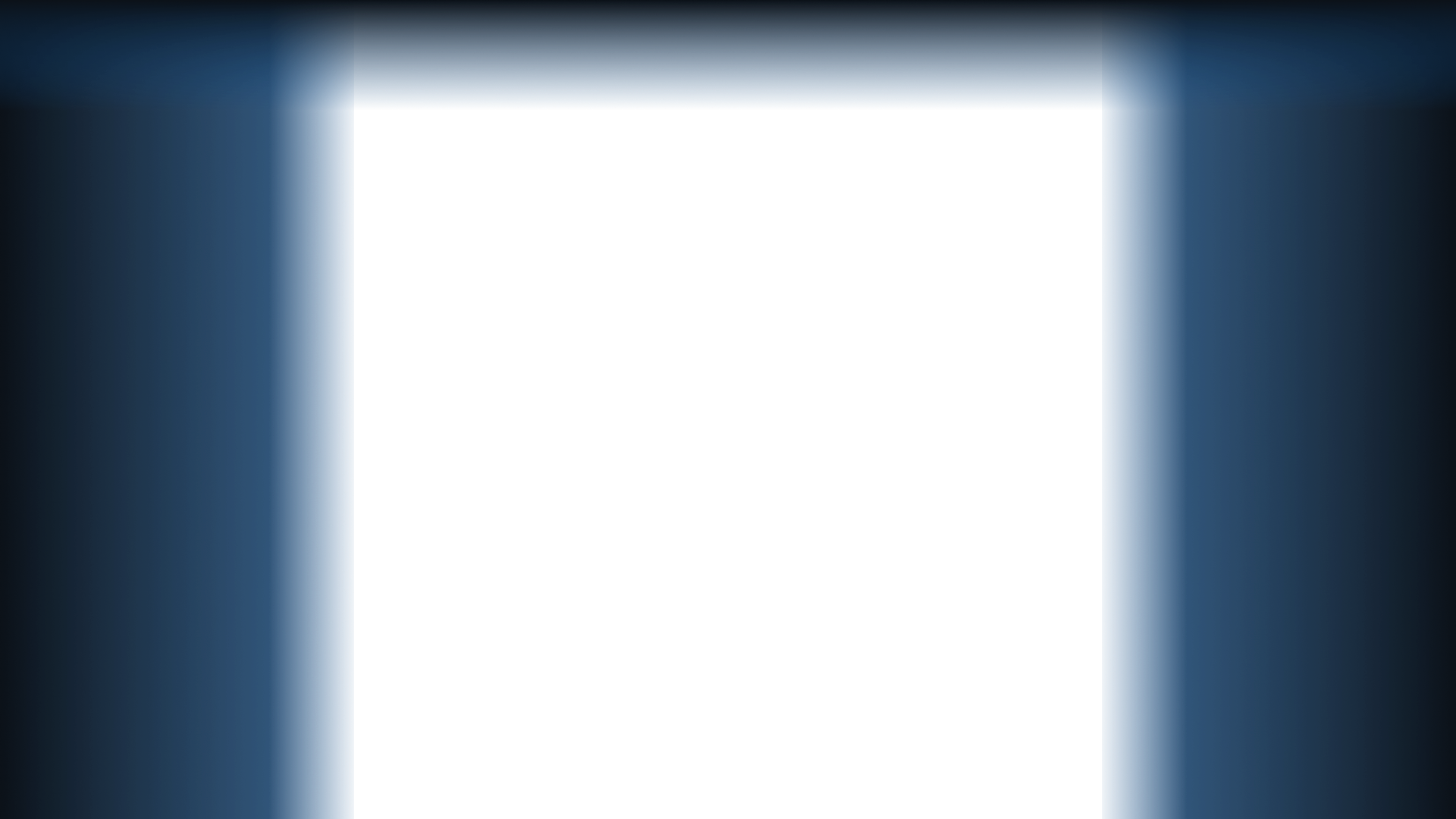

Supplement: S2 File — The original code is for Web of the platform. (ZIP) [file pone.0343700.s002.zip › zttcglweb/src/assets/map-mask.png]

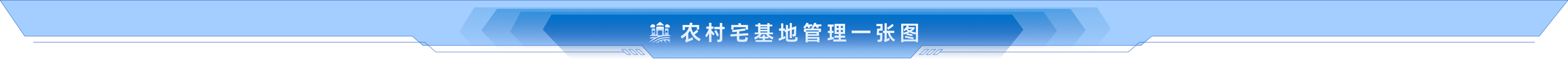

Supplement: S2 File — The original code is for Web of the platform. (ZIP) [file pone.0343700.s002.zip › zttcglweb/src/assets/map-title.png]

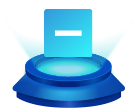

Supplement: S2 File — The original code is for Web of the platform. (ZIP) [file pone.0343700.s002.zip › zttcglweb/src/assets/minus.png]

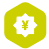

Supplement: S2 File — The original code is for Web of the platform. (ZIP) [file pone.0343700.s002.zip › zttcglweb/src/assets/money.png]

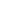

Supplement: S2 File — The original code is for Web of the platform. (ZIP) [file pone.0343700.s002.zip › zttcglweb/src/assets/password.png]

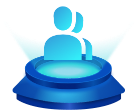

Supplement: S2 File — The original code is for Web of the platform. (ZIP) [file pone.0343700.s002.zip › zttcglweb/src/assets/people.png]

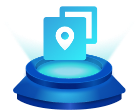

Supplement: S2 File — The original code is for Web of the platform. (ZIP) [file pone.0343700.s002.zip › zttcglweb/src/assets/position.png]

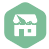

Supplement: S2 File — The original code is for Web of the platform. (ZIP) [file pone.0343700.s002.zip › zttcglweb/src/assets/shop.png]

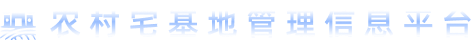

Supplement: S2 File — The original code is for Web of the platform. (ZIP) [file pone.0343700.s002.zip › zttcglweb/src/assets/title.png]

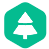

Supplement: S2 File — The original code is for Web of the platform. (ZIP) [file pone.0343700.s002.zip › zttcglweb/src/assets/tree.png]

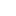

Supplement: S2 File — The original code is for Web of the platform. (ZIP) [file pone.0343700.s002.zip › zttcglweb/src/assets/user.png]

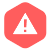

Supplement: S2 File — The original code is for Web of the platform. (ZIP) [file pone.0343700.s002.zip › zttcglweb/src/assets/warning.png]
