# Supplementary material for: Design and implementation of a comprehensive management platform for drilling engineering
Source: PLoS One. 2026 Feb 26;21(2):e0343700. doi: 10.1371/journal.pone.0343700 (PMC12944780; doi:10.1371/journal.pone.0343700)
Supplement: S1 File — The original code is for APP of the platform. (ZIP) [file pone.0343700.s001.zip › zttcgl/src/main/resources/table/钻井液日报表.htm]

|  |  |  |  |  |  |  |  |  |  |  |
| --- | --- | --- | --- | --- | --- | --- | --- | --- | --- | --- |
| 钻井液日报表 | | | | | | | | | | |
| 井号: | |  | | | | | | | | |
| 日期: | |  |  |  |  |  |  |  |  |  |
| 井深（m）: | |  | 工程情况及钻井液处理情况 | | | | | | | |
| 岩性/层位: | |  |  | | | | | | | |
| 进尺（m): | |  |  |  |  |  |  |  |  |  |
| 纯钻时间（h）： | |  |  |  |  |  |  |  |  |  |
| 钻速（m/h）： | |  |  |  |  |  |  |  |  |  |
| 钻压（KN）： | |  |  |  |  |  |  |  |  |  |
| 转速（NM）： | |  |  |  |  |  |  |  |  |  |
| 泵压（MPa） | |  |  |  |  |  |  |  |  |  |
| 泵量（l/min）: | |  |  |  |  |  |  |  |  |  |
| 钻井液性能参数 | | | | | | | | | | |
| 取样时间 | |  |  |  |  |  |  |  |  |  |
| 取样井深（m) | |  |  |  |  |  |  |  |  |  |
| 井口温度 | |  |  |  |  |  |  |  |  |  |
| 密度（g/cm^3^) | |  |  |  |  |  |  |  |  |  |
| 漏斗粘度（s) | |  |  |  |  |  |  |  |  |  |
| HTHP℃ | 失水（ml） |  |  |  |  |  |  |  |  |  |
|  | 滤饼mm |  |  |  |  |  |  |  |  |  |
| PH值 | |  |  |  |  |  |  |  |  |  |
